# First Identification and Characterization of Porcine Enterovirus G in the United States

**DOI:** 10.1371/journal.pone.0097517

**Published:** 2014-05-13

**Authors:** Srivishnupriya Anbalagan, Richard A. Hesse, Ben M. Hause

**Affiliations:** 1 Newport Laboratories, Inc., Worthington, Minnesota, United States of America; 2 Department of Diagnostic Medicine and Pathobiology, Kansas State University, Manhattan, Kansas, United States of America; Fondazione IRCCS Policlinico San Matteo, Italy

## Abstract

*Porcine enterovirus G* (EV-G) is a member of the family *Picornavirdae*, genus *Enterovirus*. To date, eleven EV-G types (EV-G1 through EV-G11) have been identified in pigs from Asia and Europe however they have never been reported in North America. In this study, we isolated and characterized the complete genome of NP/2013/USA, an EV-G from a porcine diarrhea sample from the United States. The complete genome consists of 7,390 nucleotides excluding the 3′ poly(A) tail, and has an open reading frame that encodes a 2,169 amino acid polyprotein. NP/2013/USA was most similar at the nucleotide (84%) and amino acid (95%) level to the HM131607, an EV-G1 type isolated from China in 2012.

## Introduction

Porcine enteroviruses (PEV) are members of the family *Picornaviridae*, genus *Enterovirus* and were originally divided into 13 serotypes (PEV-1 to -13) [1], [2]. Further taxonomical studies led to the subdivision of PEV into three genera *Teschovirus*, *Sapelovirus*, and *Enterovirus* with porcine species porcine teschovirus (PTV), porcine sapelovirus (PSV) and porcine enterovirus B (PEV-B) [3], [4]. Recently, PEV-B has been renamed as enterovirus G (EV-G), with the prototype EV-G viruses, PEV-9 and PEV-10, reclassified as EV-G1 and EV-G2, respectively [5], [6]. To date, the species EV-G consists of eleven types, EV-G1 to EV-G11 [3], [6]–[10].

PEV infections are generally asymptomatic. However, occasionally PTV and PSV have been associated with a wide variety of clinical conditions such as polioencephalomyelitis, enteric disease, and pneumonia [2], [7], [8]. EV-G has been isolated from healthy swine fecal samples in Asia and Europe, as well as from wild boars in Hungary [6]–[10]. Recently, EV-G was isolated from pigs with diarrhea, however no association was identified between EV-G detection and the disease [6].

EV-Gs are small, non-enveloped viruses with a positive-sense single stranded RNA genome. The genome is approximately 7400–7500 nucleotides in length. It has one open reading frame (ORF), which encodes the viral polyprotein and is flanked by 5′ and 3′ untranslated regions (UTR). The 5′-UTR vary in length between 700 and 825 nucleotides while the 3′-UTR is considerably shorter [3], [7]–[13]. 5′ and 3′-UTRs play an important role in initiation of RNA transcription and translation [13], [14]. The 5′-UTR forms secondary structure by three-dimensional folding which is used in the initiation of translation [13], [14]. The 3′-UTR forms a secondary cloverleaf structure which is essential for negative strand RNA synthesis [13], [14]. The post-translational proteolytic processing of the viral polyprotein yields four structural proteins (VP1, VP2, VP3 and VP4) and seven non-structural viral proteins (2Apro, 2B, 2C, 3A, 3B, 3Cpro and 3Dpol) [13], [14].

To date there are only seven whole genomes sequences of EV-G from samples collected from Asia and Europe. EV-G has never been reported in the United States [15]. Here we report the complete genome of an EV-G virus isolated from a clinical sample submitted to Newport Laboratories, Worthington, MN.

## Materials and Methods

### Ethics Statement

EV-G used in this study was isolated from a sample submitted to Newport Laboratories for routine diagnostic testing. The sample was obtained from a naturally infected animal in the field, by qualified veterinarians, as a part of normal veterinary care and diagnostic testing procedures.

### Virus Growth

EV-G was isolated from intestinal homogenate submitted to Newport Laboratories in August, 2013, from commercial 4-week old pig with diarrhea for diagnostic testing.

Rhesus monkey kidney cells (Marc-145) (ATCC CRL-12219) were used for virus isolation. The Marc-145 cells were propagated in DMEM (Pellgro, Manassas, VA) with either plasmocin (25 mg/l) (Invivogen, San Diego, CA) or normocin (100 mg/l) (Invivogen). Confluent cells were washed 2–3 times with replacement media (DMEM (Pellgro) with trypsin (EDTA) at 5 µg/mL and either plasmocin (25 mg/l) (Invivogen, San Diego, CA) or normocin (100 mg/l) (Invivogen). The diagnostic sample, intestinal homogenate, was centrifuged at 5000×g for 10 min to remove debris. Supernatant was used to inoculate confluent, washed Marc-145 cells. The cultures were incubated at 37°C in a CO_2_ incubator and inspected daily for cytopathic effect (CPE). If CPE was noted, media was collected and analyzed for common enteric viruses (rotaviruses A, B, and C) by quantitative reverse-transcription polymerase chain reaction (RT-PCR) (data not shown).

### PTV, PSV and EV-G Detection

RNA was extracted from Marc-145 cells with 100% CPE. 1 ml of cell culture supernatant was centrifuged at 1500×g for 10 minutes. RNA was extracted from 140 µl of the supernatant using a QIAamp Viral RNA kit (Qiagen, Valencia, CA) according to manufacturer’s instructions. RNA was eluted in 35 µl of the supplied elution buffer.

PTV, PSV and EV-G were detected by one step RT-PCR reactions using primers sets published in [15], [16]. 38-F (5′-TTGAGGATCCTCCGGCCCCTGAATGCG-3′) and 37-R primers (5′-ATCTAAGCTTGTCACCATAAGCAGCCA-3′) were used for EV-G. 1222-F (5′-GTGGCGACAGGGTACAGAAGAG-3′) and 1223-R (5′-GGCCAGCCGCGACCCTGTCAG-3′) primers were used for PTV and PSV. Amplified products were visualized after separation on 1.5 % agarose gels (TBE) containing ethidium bromide.

### RNA Isolation for Next Generation Sequencing

Marc-145 cells that showed 100% CPE following virus infection were used for RNA extraction. 20 ml of cell culture supernatant was filtered using the 0.2 µm bottle top filters (Thermo Scientific, Lenexa, Kansas). The filtrate was centrifuged at 50,000×g for 2 hrs. Supernatant was discarded and the pellet was suspended in 1000 µl of water. Samples were concentrated to a final 100 µl volume using Amicon ultra centrifugal filters (0.5 ml; 50 KDa) (Millipore, Tullagreen, Ireland). Cellular DNA and RNA were removed by incubation with DNase I (25 units) (New England Biolabs, NEB, Ipswich, MA) and RNase A (25 units) (Qiagen, Valencia, CA) at 37°C for 1 hr. RNA was extracted using Trizol LS Reagent (Life Technologies, Grand Island, NY) according to manufacturer’s instructions. The pellet containing RNA was resuspended in 20 µl of sterile H_2_O.

### Sequencing and Data Analysis

10 µg of total RNA was depleted of ribosomal RNA using GeneRead rRNA depletion kit (Qiagen) and RNA sequencing libraries were generated using the Ion Total RNA-seq kit v2 (Ion Torrent, Life Technologies) according to manufacturer’s instructions. Sequencing was carried out using Ion Personal Genome Machine (PGM) sequencing platform (Life Technologies, Grand Island, NY) as previously described [17]. Sequence reads were assembled into contigs using the SeqMan NGen program (DNAstar, Madison, WI). Sequence alignments and phylogenetic analyses were performed using MEGA 5.05 [18]. Phylogenetic comparisons were conducted using maximum likelihood analysis. Bootstrap confidence values were determined using 1000 replicates. Amino acid and nucleotide similarities were determined by using Jotun-Hein method in MegAlign program (DNAstar, Madison, WI). The EV-G was designated NP/2013/USA and submitted to GenBank (KF985175). The deep sequencing dataset was added to the Sequence Read Archive under accession SRP040697.

## Results and Discussion

### Viral Isolation

Newport Laboratories received an intestinal homogenate sample from a pig with diarrhea for diagnostic testing. Samples were positive for group A rotavirus (Ct = 33) and negative for groups B and C rotavirus by one step RT-PCR (data not shown). Subsequently, isolation of group A rotavirus was attempted on MARC-145, however, was unsuccessful as determined by one step RT-PCR despite CPE suggestive of virus propagation. Samples were next tested for PTV, Sapelovirus (PEV-8), and EV-G by one step RT-PCR (data not shown). The samples were positive only for EV-G. Further deep sequencing identified viral reads mapping soley to EV-G.

### Sequence Analysis

The NP/2013/USA genome was sequenced with an Ion Torrent Personal Genome Machine. The complete genome contained 7,390 nucleotides (nt) excluding the 3′ poly(A) tail. The 5′-UTR consisted of 811 nucleotides and the 3′-UTR was comprised of 72 nucleotides. A single large open reading frame was identified from nucleotides 812 to 7318 and encoded a hypothetical 2,169 amino acid polyprotein. The genome had 28.3% A, 24.9% T, 24% G, and 22.9% C which is similar to the other EV-G isolates. The genome organization of NP/2013/USA was identical to the CPE group III strains, now classified as EV-G [4], [8], [9], [19].

**Figure 1:**
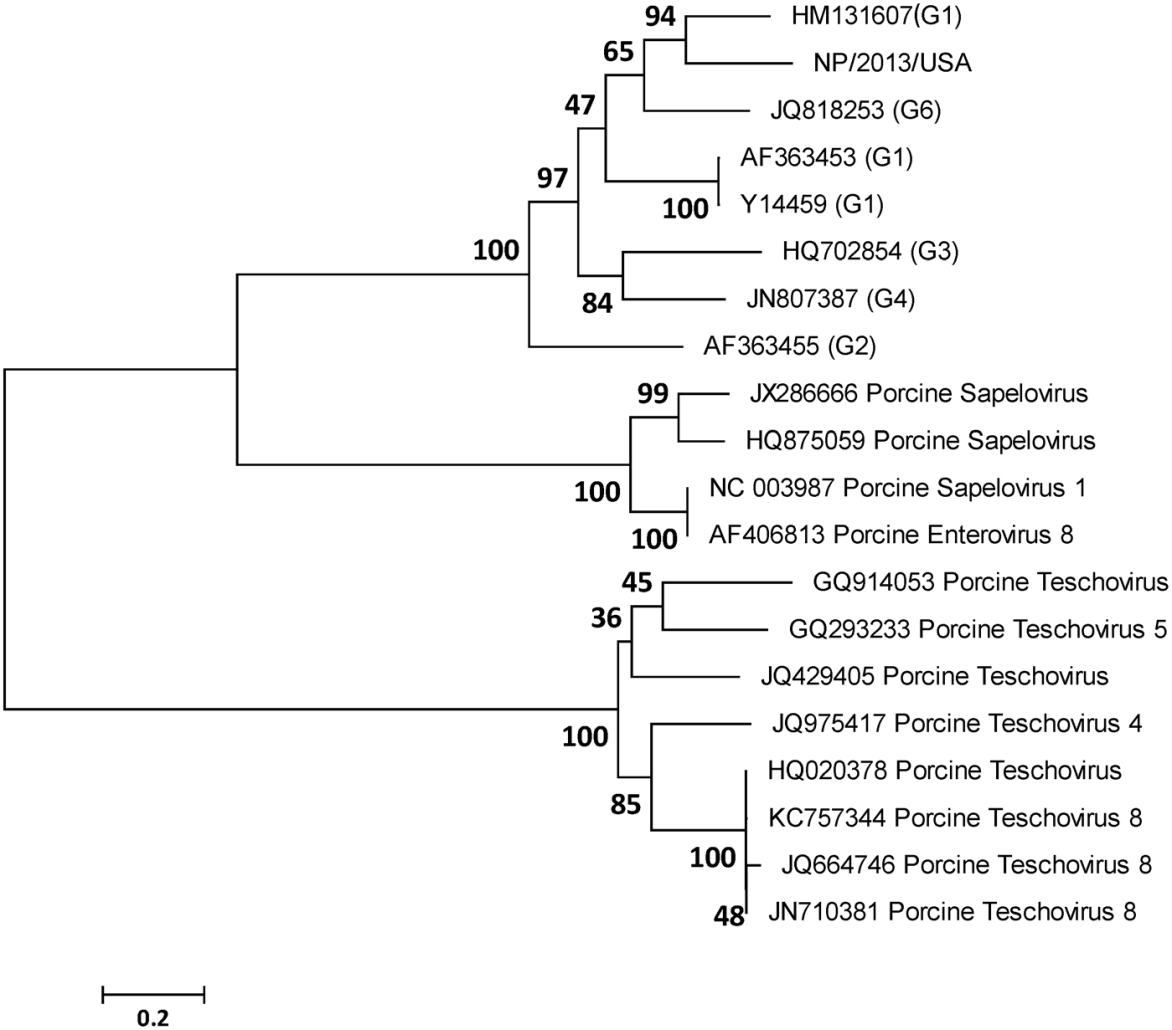
Phylogenetic tree of NP/2013/USA with *Enterovirus G*, *Teschovirus* and *Sapelovirus* genomes. Maximum-likelihood analysis in combination with 1000 bootstrap replicates was used to derive trees based on the nucleotide sequence in the whole genome. A scale representing the number of nucleotide changes is shown. Bootstrap values are shown to the left of major nodes.

To elucidate the genetic relatedness of NP/2013/USA with PTV, PSV, and EV-G types, a phylogenetic tree was constructed using the maximum likelihood algorithm and bootstrapped using 1000 replicates using the complete genome sequence. NP/2013/USA segregated into the same clade as other EV-G and was clearly distinct from PTV and PSV (Fig. 1). NP/2013/USA was most closely related to EV-G.

NP/2013/USA showed overall nucleotide similarities of 84, 81, 80, 80, 75, 77, and 77% respectively to HM131607 (EV-G1), JQ818253 (EV-G6), AF363453 (EV-G1), Y14459 (EV-G1), AF363455 (EV-G2), HQ702854 (EV-G3), and JN807387 (EV-G4) strains respectively (Table 1), and 95, 90, 82, 90, 82, 87, and 86% polyprotein amino acid sequence similarities, respectively.

**Table 1:** Nucleotide identity (%) of NP/2013/USA with EV-G isolates in GenBank.

Accession number	HM131607	JQ818253	AF363453	Y14459	AF363455	HQ702854	JN807387
PEV type	EV-G1	EV-G6	EV-G1	EVG-1	EV-G2	EV-G3	EV-G4
**overall coding**	84	81	80	80	75	77	77
**5′NCR**	89	91	91	89	87	89	86
**3′NCR**	99	97	94	94	97	94	80
**1A(VP4)**	80	80	80	80	76	82	80
**1B(VP2)**	80	72	76	76	70	71	71
**1C(VP3)**	79	73	76	76	71	70	72
**1D(VP1)**	77	68	74	74	65	69	67
**2A(pro)**	82	82	83	83	83	79	80
**2B**	86	87	81	81	75	80	84
**2C**	84	87	80	80	75	80	80
**3A**	85	84	75	75	76	77	80
**3B**	77	74	70	70	76	81	73
**3C(pro)**	85	84	80	80	80	79	80
**3D(pol)**	88	87	84	84	80	82	82

The most variable region, as well as the antigenic determinant, VP1 showed nucleotide similarities of 77, 68, 74, 74, 65, 69, and 67% to HM131607 (EV-G1), JQ818253 (EV-G6), AF363453 (EV-G1), Y14459 (EV-G1), AF363455 (EV-G2), HQ702854 (EV-G3), and JN807387 (EV-G4) strains respectively (Table 1), and VP1 amino acid sequence similarities of 88.5, 70.5, 79.5, 79.9, 62.3, 66, and 68% respectively. The various EV-G types share >25% and >15% nucleotide and amino acid divergence respectively from each other (http://www.picornaviridae.com). The high amino acid homology of NP/2013/USA to EV-G1 suggests that it should be classified as type G1 (Table 1).

Enteroviruses utilize recombination as a mechanism for evolution, with genetic diversification principally in genes encoding structural proteins [20]. Genetic analysis of NP/2013/USA suggests a similar pattern of recombination. The 5′-UTR, 3′-UTR, and non-structural genes had 89%, 99% and 85% nucleotide identity to a 2012 Chinese EV-G1 isolate (HM131607). In contrast, the region encoding the structural proteins had only 79% identity to the same virus. Previously, recombination was described to occur in the region involving the 5′-UTR [7]. However, recent studies suggested that sequence differences in the 5′-UTR are the result of the accumulation of mutations but not a recombination event [6]. Furthers studies by Nguyen et al. indicate that EV-G types circulating in Europe and Asia share a common 5′-UTR sequence with the prototype EV-G1 (AF363453) and –G2 (AF363455) isolated in 1973 and 1975 respectively [6]. With only 89% nucleotide identity to previously sequenced EV-G, it is unclear whether the 5′UTR of NP/2013/USA is derived via recombination.

EV-G infection was detected at higher frequencies in younger pigs than in adults from Vietnam, Czech Republic, China, Hungary, Italy, and Spain [6], [12], [21]–[23]. EV-G1, 6, and 8–11 types were found in pigs of ages 3–9 weeks, 7–9 months, and 52–165 months respectively [6]. Similar age associated differences in infection frequencies of enterovirus serotypes were identified in young children [24]. NP/2013/USA, an EV-G1 type, was isolated from nursery pigs (3–10 weeks of age) similar to other EV-G1 [6]. While NP/2013/USA was isolated from pigs with diarrhea, group A rotavirus was also present in the fecal samples. The contribution of each virus to clinical disease is unclear however group A rotaviruses have been well established as pathogens of pigs with diarrhea the most common clinical presentation. A controlled pig inoculation experiment is needed to ascertain the pathogenic potential of NP/2013/USA.

In conclusion, the present study was the first to identify EV-G in pigs in the United States. NP/2013/USA’s nucleotide and protein sequence was most homologous to HM131607, an EV-G1 isolate from China in 2012. It is unclear whether our finding of EV-G in U.S. pigs represents a recent introduction of this virus to the U.S. or whether EV-G has circulated in U.S. herds undetected for some time. Previous work has failed to find EV-G in U.S. pigs [15]. Also of interest is the recent introduction of porcine epidemic diarrhea virus (PEDV) into the U.S. swine herd [25]. Massive outbreaks of PED occurred throughout the U.S. in 2013 following the introduction of this foreign virus. Interestingly, the PEDV epidemic in the U.S. is genetically nearly identical to a Chinese PEDV isolated in 2012 [26], [27]. It is unclear whether the isolation of a second virus not previously seen in the U.S. with high genetic similarity to contemporary Chinese viruses is coincidental or due to a similar recent introduction. Serological testing of contemporary and historic samples is needed to clarify the epidemiology of EV-G in the U.S.

Disclaimer: This document is provided for scientific purposes only. Any reference to a brand or trademark herein is for informational purposes only and is not intended for a commercial purpose or to dilute the rights of the respective owner(s) of the brand(s) or trademark(s).
